# Transgenerational Effects of Prenatal Endocrine Disruption on Reproductive and Sociosexual Behaviors in Sprague Dawley Male and Female Rats

**DOI:** 10.3390/toxics10020047

**Published:** 2022-01-20

**Authors:** Bailey A. Kermath, Lindsay M. Thompson, Justin R. Jefferson, Mary H. B. Ward, Andrea C. Gore

**Affiliations:** 1 Institute for Neuroscience, The University of Texas at Austin, Austin, TX 78712, USA; baileykermath@gmail.com; 2 Division of Pharmacology & Toxicology, College of Pharmacy, The University of Texas at Austin, Austin, TX 78712, USA; lindsay.thompson82@utexas.edu (L.M.T.); justin.r.jefferson@gmail.com (J.R.J.); maryhbw@gmail.com (M.H.B.W.)

**Keywords:** endocrine-disrupting chemical (EDC), polychlorinated biphenyl (PCB), Aroclor 1221 (A1221), transgenerational, social behavior, mating behavior, paced mating, ultrasonic vocalization (USV), estradiol

## Abstract

Endocrine-disrupting chemicals (EDCs) lead to endocrine and neurobehavioral changes, particularly due to developmental exposures during gestation and early life. Moreover, intergenerational and transgenerational phenotypic changes may be induced by germline exposure (F2) and epigenetic germline transmission (F3) generation, respectively. Here, we assessed reproductive and sociosexual behavioral outcomes of prenatal Aroclor 1221 (A1221), a lightly chlorinated mix of PCBs known to have weakly estrogenic mechanisms of action; estradiol benzoate (EB), a positive control; or vehicle (3% DMSO in sesame oil) in F1-, F2-, and F3-generation male and female rats. Treatment with EDCs was given on embryonic day (E) 16 and 18, and F1 offspring monitored for development and adult behavior. F2 offspring were generated by breeding with untreated rats, phenotyping of F2s was performed in adulthood, and the F3 generation were similarly produced and phenotyped. Although no effects of treatment were found on F1 or F3 development and physiology, in the F2 generation, body weight in males and uterine weight in females were increased by A1221. Mating behavior results in F1 and F2 generations showed that F1 A1221 females had a longer latency to lordosis. In males, the F2 generation showed decreased mount frequency in the EB group. In the F3 generation, numbers of ultrasonic vocalizations were decreased by EB in males, and by EB and A1221 when the sexes were combined. Finally, partner preference tests in the F3 generation revealed that naïve females preferred F3-EB over untreated males, and that naïve males preferred untreated over F3-EB or F3-A1221 males. As a whole, these results show that each generation has a unique, sex-specific behavioral phenotype due to direct or ancestral EDC exposure.

## 1. Introduction

Endocrine-disrupting chemicals (EDCs) interfere with hormone action within an organism [1,2]. These chemicals, or mixture of chemicals, act upon the neuroendocrine systems that govern physiological processes such as reproduction, immune function, metabolism, and sex-typical behaviors in adulthood. Exposure to environmental EDCs during critical periods of development such as gestation can alter the organization of these neuroendocrine systems and predispose organisms towards disease and maladaptive traits. Known as the Developmental Origins of Health and Disease or DOHaD [3], this phenomenon has been well studied for a variety of health outcomes in individuals who experienced direct exposure early in life (F1 generation). Regarding neuroendocrine functions and hormone-dependent behaviors, the focus of this study, exposures to EDCs including bisphenol A (BPA), phthalates, and persistent organic pollutants such as polychlorinated biphenyls (PCBs) induce adverse phenotypic outcomes in animal studies [4,5,6,7,8,9,10,11,12,13,14,15,16,17], and are associated with increased prevalence of neurobehavioral disorders in epidemiological studies in humans [18,19,20,21,22].

EDCs also exert actions on the F2 generation, exposed as germ cells within the F1 embryo. The F3 generations and beyond can exhibit phenotypic changes in the absence of direct exposure, presumably through germline epigenetic inheritance [23,24]. Although few in number, studies on inter- and transgenerational effects of EDCs have reported sexually dimorphic effects on behaviors, especially those influenced by early life endogenous hormones ([16,25,26,27,28,29,30,31,32]; reviewed in [33]). More research comparing generational effects is needed to better understand how legacy chemicals that are no longer actively manufactured but are still persistent in the environment, such as PCBs, may lead to heritable effects generations later.

The current study aims to build upon previous studies in the lab that identified transgenerational effects of PCBs on physiology, behavior, and hypothalamic gene expression throughout development [29,30,34,35]. Here, we extend these findings by examining mating behavior and sociosexual ultrasonic vocalization and partner preference activity in the F1, F2 and F3 generations to show sex- and generation-specific disruption in adult female and male rats.

## 2. Materials and Methods

### 2.1. Experimental Design and Animal Husbandry

All animal protocols were conducted in accordance with NIH and USDA guidelines and were approved by the Institutional Animal Care and Use Committee (IACUC) at The University of Texas at Austin. Sprague Dawley rats were obtained from Harlan Laboratories (Houston, TX, USA), switched to the low-phytoestrogen Harlan-Teklad 2019 Global Diet ad libitum, and housed in same-sex groups (2–3 per cage) under constant humidity and temperature (21–22 °C) and a partially reversed 12:12 L:D cycle (lights on at 2400 h). Virgin females were impregnated in house. The morning after a sperm-positive vaginal smear was termed embryonic day (E) 1. On E16 and E18, during the period of sexual differentiation of the brain, F0 dams were weighed and randomly injected with one of three treatment groups: 1 mg/kg Aroclor 1221 (A1221, an estrogenic PCB mixture, administered intraperitoneally [i.p.]), 50 μg/kg estradiol benzoate (EB; administered subcutaneously [s.c.]), or a negative vehicle control (3% DMSO in sesame oil, injected i.p. or s.c., and combined into one DMSO group). Dosages and routes were selected to be identical to other studies in our lab and to be human relevant [35,36,37,38,39]. F0 litters were spread over 6 cohorts for a total of: DMSO, *n* = 14; EB, *n* = 11; A1221, *n* = 12.

**Figure 1:**
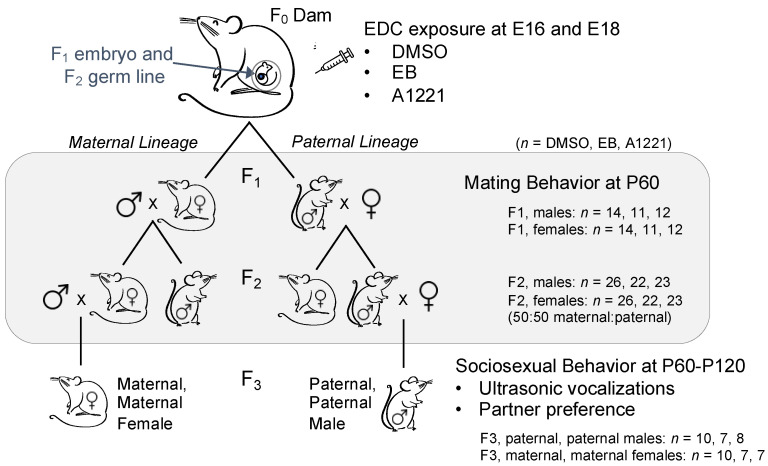
The transgenerational experimental design. Abbreviations: EDC: endocrine-disrupting chemical, E: embryonic day, DMSO: dimethyl sulfoxide, EB: estradiol benzoate, A1221: Aroclor 1221, and P: postnatal day. Gray shading indicates those generations used in the current study for mating behaviors. The F3 generation was used for sociosexual behaviors.

Behavioral and physiological reproductive endpoints were examined after rats reached sexual maturity, using 1 male and female from each litter (Figure 1). F1 males and females were examined for sexual behaviors as young adults (P60) while mated to naïve rats (purchased from Harlan). After behavioral testing, F1 females carried litters to term. F2 offspring were also observed for sexual behavior during mating at P60 and the pregnant F2 dams carried the F3 generation to term. Finally, F3 maternal-maternal lineage females and paternal-paternal lineage males were examined for adult sociosexual behaviors (P60–120). A set of untreated rats (UNT, *n* = 6) were raised in the lab alongside the F3 offspring as an additional negative control group, in which dams were restrained and finger-poked to simulate an injection. Harlan-raised males and females used for F3 sociosexual experiments were received at 2 months of age and allowed to acclimate to the lab for 3–4 weeks before experimentation.

### 2.2. Tissue Collection

Males and female rats were euthanized between P113–127. For all rats, adrenals and gonads were removed, weighed, and normalized to body weight. Trunk blood was collected from F1 and F2 rats, allowed to clot and spun at 1500× *g* for 5 min. Serum was separated and stored at −80 °C until further analysis.

### 2.3. Serum Hormone Assays

F1- and F2-generation serum samples were used to investigate the concentrations of circulating testosterone (males) and estradiol (females). Concentrations of serum testosterone were detected in duplicate using an RIA kit, as recommended by the manufacturer (Cat. No. 07189102, MP Biomedicals, Santa Ana, CA, USA). The assay range was 0.1–10 ng/mL, assay sensitivity 0.03 ng/mL and intra-assay variability 1.8%. Serum estradiol samples were run in duplicate using the estradiol RIA kit (Cat. No. DSL-4800, Beckman Coulter, Brea, CA, USA). The assay range was 5–720 pg/mL, assay sensitivity 2.2 pg/mL and intra-assay variability 3.0%.

### 2.4. Ovariectomy and Hormone Priming for Sociosexual Experiments

Stimulus females used in the ultrasonic vocalization testing were ovariectomized. During surgery, an estradiol Silastic capsule was placed s.c. between the shoulder blades. After recovery, these rats received a s.c. dose of 590 μg progesterone 4 h prior to use to induce receptivity. For the other behaviors, females remained ovarian-intact but were hormone-primed to ensure receptivity during experiments. Ovarian-intact females were given 50 μg estradiol s.c. 52 h, and 590 μg progesterone 4 h, prior to behavioral testing [32]. In all cases, receptivity was confirmed with a sexually experienced male that was otherwise not used in the experiment.

### 2.5. Reproductive Behavior and Fertility in F1 and F2 Rats

To determine whether prenatal endocrine disruption adversely affects adult reproductive behavior in the F1 and F2 generations, mating trials were conducted at P60 in a non-paced setting. F1 and F2 females were tested on the day of behavioral estrus with a sexually experienced, Harlan-purchased male. F1 and F2 males were tested with sexually naïve, Harlan females in behavioral estrus. Mating trials were performed under dim red light and videotaped for subsequent scoring. Males were acclimated for 10 min to the mating chamber (30 × 38 cm) 5 h before the trial and then returned to the same chamber for 5 min immediately before the trial start at 1600 h. The start time was recorded when the female was placed into the mating chamber. Trials only proceeded if the female was receptive and the male displayed mounting behavior within the first 20 min.

Videos were scored by an experimenter blind to treatment for the following male sexual behaviors: mount frequency, intromission frequency, latencies to mount, intromit, and ejaculate, and the postejaculatory interval (PEI). Because the experimental males were sexually inexperienced and thus slow to display mating behavior, their ejaculation latencies and PEI scores were capped at 30 min after the first mount and 15 min after ejaculation, respectively. Intromission rate was calculated as number of intromissions over the number of mounts with or without penetration. Copulatory rate was calculated as the number of mounts and intromissions from the start time until ejaculation. Female sexual behaviors scored were proceptive (hops and darts only, as ear wiggling could not be scored from the videotape), receptive (lordosis quotient, or the percentage of lordosis responses for the first 10 male mounts, and lordosis intensity score, rating the magnitude of each spinal dorsiflexion from 0 to 3, with 0 representing no spinal dorsiflexion and 3 an exaggerated dorsiflexion and head and rump elevation) and rejection (kicking, boxing, biting, escape, rolling) behaviors for the first 10 male copulatory acts. We further calculated the proceptive rate and rejection rate as the number of acts over the time scored and the latency to display the first lordotic response.

### 2.6. USV Recording in Sociosexual Context in F3 Rats

USVs were elicited in a sociosexual context for the F3 generation and recorded in a glass chamber (30 × 76 × 45 cm) equipped with an ultrasonic microphone (CM16, Avisoft Bioacoustics, Glienicke/Nordbahn, Germany), as published [6,29]. USVs were sampled at a 250 kHz sampling rate with 16-bit resolution through an A/D card (National Instruments, Austin, TX, USA) using RECORDER NA-DAQ software (v4.2.16, Avisoft Bioacoustics, Glienicke/Nordbahn, Germany). All trials were performed 1–3 h after lights off under dim red light. Experimental rats were sexually naïve, F3 EDC- and control-lineage males and females, aged P60–P120. F3 females were ovarian-intact and hormone-primed to be receptive on the final day of testing. Each experimental rat underwent three separate days of trials, following a previously validated protocol [40]. Days 1 and 2 consisted of a 10-min trial in the recording chamber to habituate the animals and obtain baseline USV recordings. On the final day, a sexually experienced stimulus rat of the opposite sex was placed into the chamber with the experimental rat, separated by a wire mesh partition. They were allowed to interact through the mesh wire for 5 min at which point the stimulus rat was removed from the room and 10 min of USVs were recorded from the experimental rat. Recorded USVs were analyzed with SASlab Pro software (v5.2.07, Avisoft Bioacoustics, Glienicke/Nordbahn, Germany), which automatically measures the number and acoustic parameters of USVs. Sonograms were generated under a 512 FFT-length and 75% overlap frame setup. As flat 50 kHz USVs may have unique communicative properties compared to calls with frequency modulation, USVs were separated into flat and frequency-modulated (FM) calls using an unbiased and replicable technique that categorizes USVs based on their bandwidth, or the maximum peak frequency minus the minimum peak frequency. Calls with a bandwidth of 5 kHz or more were classified as FM and a bandwidth of less than 5 kHz as flats (non-FM) [41,42]. The total number of 50 kHz USVs, number of FM and non-FM calls for the first 5 min of each recording session were analyzed.

### 2.7. Partner Preference in F3 Rats

F3 EDC- and control-lineage males and females were used after USV testing, approximately 4–7 h after lights off under dim red light. Partner preference trials were conducted as previously described [32]. All rats were sexually naïve and all females remained gonadally intact but were hormone primed to be receptive on the final day of testing. Trials were conducted in a glass arena (122 × 46 cm) and recorded by a video camera connected to ANY-maze software (v4, Stoelting Co., Wood Dale, IL, USA). In order to determine whether F3 EDC- or control-lineage rats could be distinguished from untreated animals in a mating-induced partner preference paradigm, we placed an F3 experimental rat (A1221-, EB-, DMSO-lineage or UNT) opposite a Harlan rat as the Stimulus rats. After Stimulus rats were placed on opposing sides of the arena, a Chooser rat of the opposite sex was allowed to explore and interact with the stimulus rats through wire mesh dividers [32]. Harlan-raised males and females were used as the Choosers and were a separate set from those used as stimulus animals.

Chooser rats were habituated to the empty arena in a 10-min trial on days 1 and 2. On day 3, Stimulus rats were placed behind opposite wire mesh dividers and allowed to acclimate for 5 min. Next, the Chooser rat was placed in the center of the arena and given 10 min to explore and interact with the Stimulus rats across the wire mesh. Trials were repeated up to three times, in which the location and identity of the stimulus rats were exchanged to avoid confounding biases. Behaviors (grooming, rearing, facial investigation, contact with Plexiglas dividers, speed) and total time and total time active (the combination of all scored behaviors) spent in each zone were scored by an experimenter blind to treatment and analyzed by ANY-maze software (v4, Stoelting Co., Wood Dale, IL, USA). Behavior from the area immediately surrounding the wire divider of the stimulus rat (the wire zone) was used for analysis. Data from the Harlan stimulus rat were subtracted from the F3-lineage rat to calculate a preference score in which positive numbers indicate more time spent near the F3-lineage rat.

### 2.8. Statistical Analysis

Data were analyzed with R 4.1.0 [43], the rstatix [44], the emmeans [45], the lme4 [46], the lmerTest [47], and the ARTool [48,49,50] packages. Scores over 2.5 standard deviations were considered outliers and removed from the analysis. When outliers were present, only one outlier was detected and removed per group with the one exception of the number of proceptive behaviors in the female F2-DMSO group, in which two outliers were removed. Outliers were distributed evenly across groups. Maternal and paternal lines in the F2 generation were combined for statistical analysis as parental lineage did not significantly impact the endpoints examined. For all somatic (F1, F2 and F3 generations) and mating behavior (F1 and F2 only) outcomes, a one-way analysis of variance (ANOVA) was run for Treatment. Kruskal–Wallis tests were used when data did not meet the Levene’s homogeneity of variance or Shapiro–Wilk normality tests. Holm–Sidak or Dunn pairwise post hoc comparisons were run when a significant main effect was found. USV parameters were analyzed with a two-way ANOVA for Sex and Treatment. If data did not meet ANOVA assumptions, even after attempts of data transformation techniques, we used the Aligned Rank Transform (ART) for non-parametric factorial ANOVA [49,50] and the corresponding ART-C pairwise post hoc comparisons [48]. Finally, wire zone preference scores from the partner preference test were run separately for males and females using a linear mixed model with F0 treatment as a Fixed Variable, Animal ID as a Random Variable, and Trial Number as a Repeated Variable. For all data, alpha was set to 0.05.

## 3. Results

A summary of statistically significant results is provided in Table 1.

### 3.1. Transgenerational Somatic Changes

#### 3.1.1. Males

**Figure 2:**
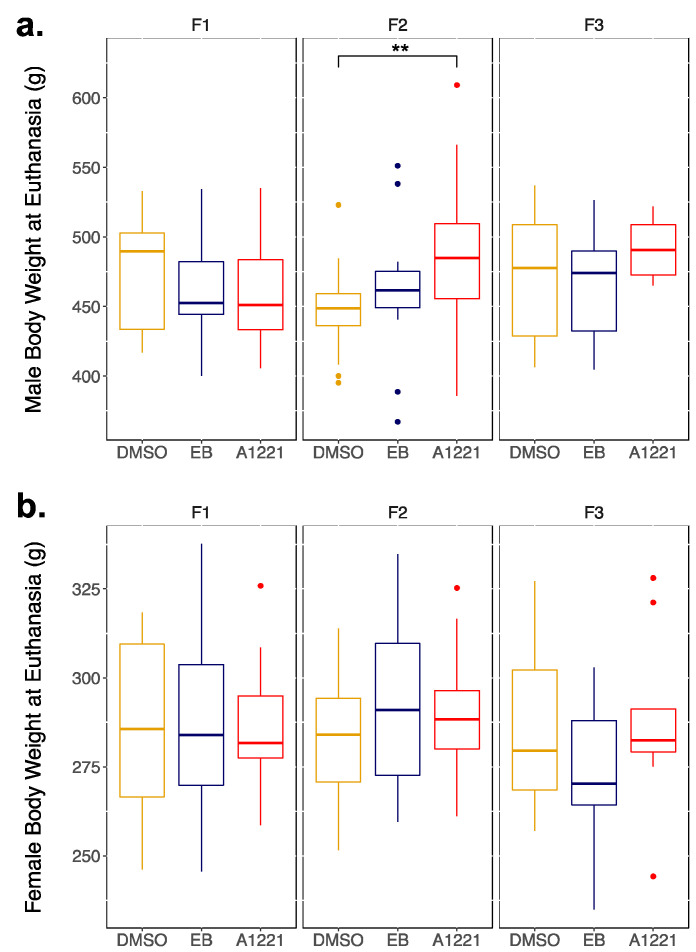
Boxplots of body weight at euthanasia (~P120) for adult (**a**) males and (**b**) females. Data were analyzed by one-way ANOVA or Kruskal–Wallis for effect of F0 treatment, followed by Holm–Sidak or Dunn’s pairwise comparisons. ** *p* < 0.01. In this and other boxplot graphs, the line represents the median, the lower and upper outline of the boxes the 25th and 75th percentile, respectively, and the lines the 95th percentile.

Few somatic changes were detected in the measured outcomes for EDC-lineage rats. A trend was observed for an F0 treatment effect (EB slightly larger than DMSO) in normalized adrenal weights of F1 (F(2,33) = 2.997, *p* = 0.064) and F3 (F(2,25) = 2.856, *p* = 0.076) males (Table 2). Similarly, no changes were detected in serum testosterone levels (F3 hormones not measured) or normalized testes weight. However, in the F2 generation, we observed a significant effect of treatment on male body weight (H(2) = 9.054, *p* = 0.011) with A1221-lineage males having greater average body weights compared to DMSO controls (*p* = 0.008; Figure 2a).

#### 3.1.2. Females

In females, we found an effect of F0 treatment on normalized uterine weight in the F2 generation (H(2) = 6.434; *p* = 0.040), in which A1221-lineage females had greater uterine weights compared to EB (Dunn’s post hoc, *p* = 0.044; Table 2). No effect was found for female body weight (Figure 2b), normalized adrenal weight, normalized ovarian weight or serum estradiol (F3 hormones not measured). Hormone priming for the sociosexual tests also resulted in an expected increase in normalized ovarian and uterine weights in the F3 generation, regardless of F0 treatment (Table 2).

### 3.2. Reproductive Behavior in the F1 and F2 Generations

#### 3.2.1. Males

**Figure 3:**
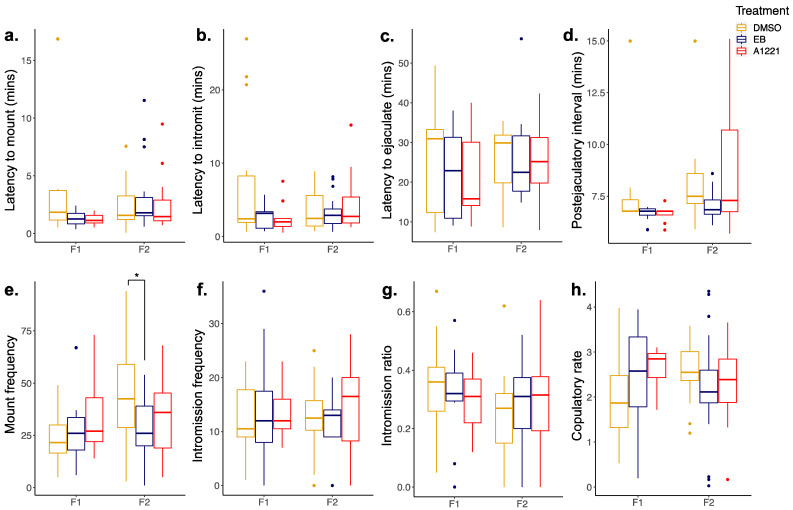
Boxplots of male mating behavior at P60 for F1 and F2 generations. Sexually naïve males were scored for (**a**) latency to mount, (**b**) latency to intromit, (**c**) latency to ejaculate, (**d**) postejaculatory interval, (**e**) mount frequency, (**f**) intromission frequency, (**g**) intromission ratio (calculated as number of intromissions divided by number of mounts), and (**h**) copulatory rate (calculated as the number of mounts and intromissions from the start time until ejaculation). Data were analyzed by one-way ANOVA or Kruskal–Wallis for effect of F0 treatment, followed by Holm–Sidak or Dunn’s pairwise comparisons. F1: *n* = 14 DMSO, 11 EB, 11 A1221; F2: *n* = 26 DMSO, 22 EB, 22 A1221. * *p* < 0.05.

Overall, few effects of perinatal EDC treatment were found in male mating behavior (Figure 3). In the F2 generation, a significant effect of treatment was found for mount frequency (F(2,66) =3.374; *p* = 0.035). Holm–Sidak post hoc analysis showed that EB-lineage males had a lower mount frequency compared to DMSO (*p* = 0.035; Figure 3e) suggesting that EB males required fewer mounts to reach ejaculation. However, no changes were observed in intromission or ejaculation behaviors (Figure 3). All treatments groups showed long average ejaculation latencies with high variability within the groups, presumably due to male subjects being sexually naïve at the time of testing.

#### 3.2.2. Females

**Figure 4:**
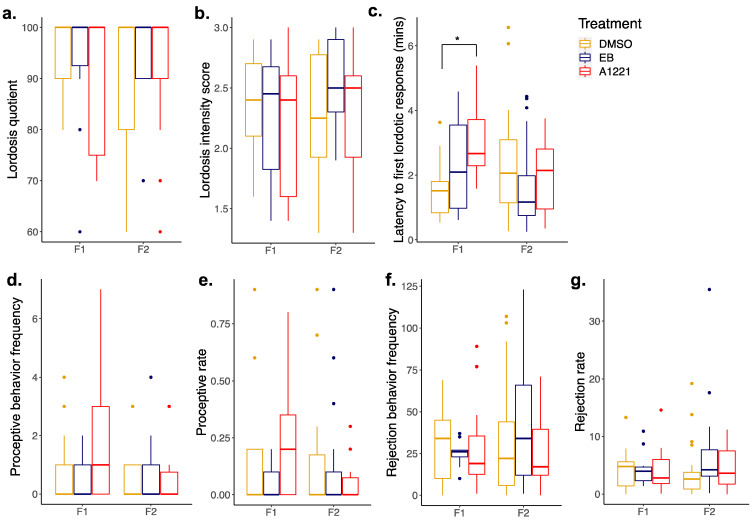
Boxplots of female mating behavior at P60 for F1 and F2 generations. Sexually naïve females were scored for (**a**) lordosis quotient, (**b**) lordosis intensity score, (**c**) latency to first lordosis response, (**d**) proceptive behavior frequency, (**e**) proceptive rate, (**f**) rejection behavior frequency and (**g**) rejection rate. Data were analyzed by one-way ANOVA or Kruskal–Wallis for effect of F0 treatment, followed by Holm–Sidak or Dunn’s pairwise comparisons. F1: *n* = 14 DMSO, 11 EB, 12 A1221; F2: *n* = 23 DMSO, 22 EB, 23 A1221. * *p* < 0.05.

Sexually naïve F1 and F2 females were examined for copulatory, proceptive and receptive behaviors (Figure 4). All females were in behavioral estrus during mating trials and were successfully able to lordose in response to male mounting and intromitting behavior. In F1 females, the latency to display the first lordotic response was affected by treatment (H(33) = 3.83; *p* = 0.032). Post hoc analysis revealed A1221-exposed females had significantly longer latencies compared to DMSO-exposed females (*p* = 0.015) despite mounting attempts by a sexually experienced male (Figure 4c). Overall, females displayed high levels of aversive behavior and few proceptive behaviors, likely due to the non-paced setting of the mating trials.

### 3.3. Sociosexual Behaviors in the F3 Generation

#### 3.3.1. Ultrasonic Vocalizations (USVs)

**Figure 5:**
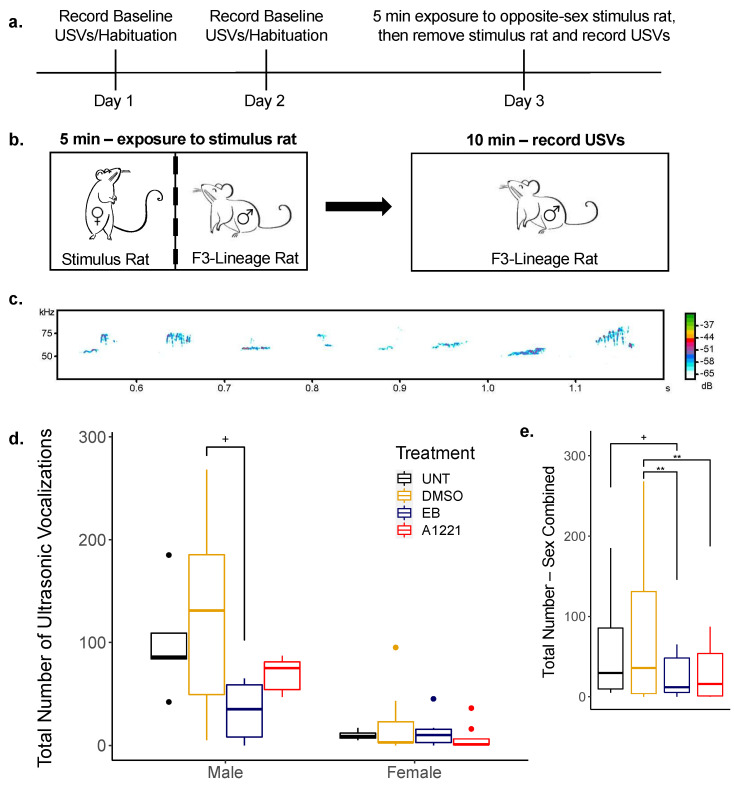
Ultrasonic vocalizations (USVs) emitted by F3−generation males and females (P60–120) in response to an opposite−sex rat. An untreated control group (UNT) was raised across generations in−house alongside the F3 litters. (**a**) Timeline of USV experiment; (**b**) Diagram of experiment on day 3; (**c**) example spectrogram of recorded USVs; (**d**) Boxplots of the total number of USV calls (frequency modulated [FM] and non−FM) during the first 5 min of recording by sex; (**e**) Boxplots of the total number of USV calls with sex combined. Data were analyzed by two−way ANOVA or Aligned Rank Transformation (ART) for effect of F0 treatment and sex, followed by Holm−Sidak or ART−C pairwise comparisons. Males: *n* = 6 UNT, 10 DMSO, 6 EB, 6 A1221; females: *n* = 5 UNT, 9 DMSO, 6 EB, 8 A1221. + *p* < 0.07; ** *p* < 0.01.

We examined the number and duration of appetitive 50 kHz USVs in F3 adults within a mating context (Figure 5). Experimental females were ovarian-intact but hormone primed to ensure receptivity during testing. To reduce potential variability caused by mixed maternal vs. paternal lineages, only maternal, maternal F3 females and paternal, paternal F3 males were used (see Figure 1). Two-way ANOVA tests revealed significant sex and treatment effects in USVs within the first 5 min of separation from the stimulus rat. In particular, the total call number was significantly affected by sex (F(1,46) = 34.96; *p* < 0.001), F0 treatment (F(3,46) = 6.80; *p* < 0.001) and their interaction (F(3,46) = 5.72; *p* = 0.002; Figure 5c). Post hoc treatment contrasts revealed a trend for EB-lineage males to call less frequently than DMSO controls (*p* = 0.065). When sexes were combined to further examine the treatment main effect, we found that DMSO-lineage controls emitted more USVs than both EB (*p* = 0.003) and A1221 (*p* = 0.007) groups (Figure 5e). There was also a trend for reduced call number between EB-lineage rats and our in-house bred untreated controls (UNT; *p* = 0.063).

Similar effects were seen when analyzing two subtypes of USVs: frequency-modulated (FM) and non-FM calls (Table 3). Males emitted both types of calls more frequently (non-FM: F(1,42) = 31.87, *p* < 0.001; FM: F(1,41) = 19.89, *p* < 0.001) and called for longer average durations (F(1,44) = 4.932, *p* = 0.032) than females. The number of non-FM calls was also affected by F0 treatment (F(3,42) = 2.79, *p* = 0.024), with EB males having fewer calls of this subtype than DMSO (*p* = 0.031).

#### 3.3.2. Partner Preference

**Figure 6:**
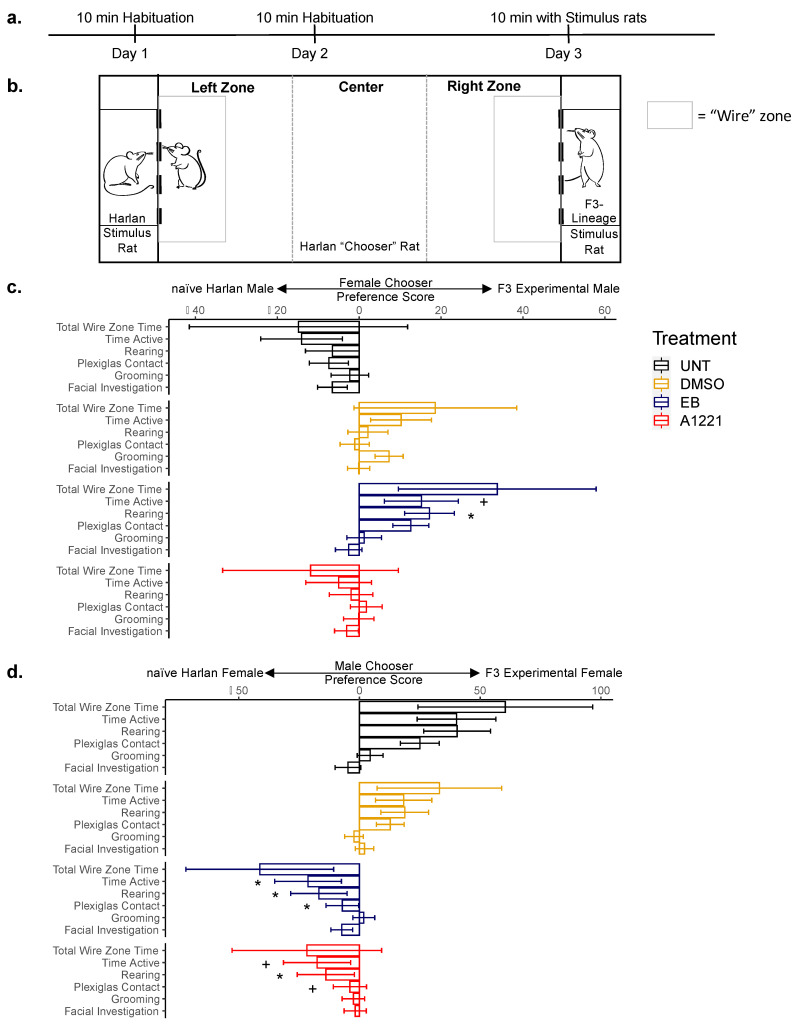
Partner preference (PP) in a mating context by F3-generation males and females (P60–120). The Chooser was a naïve rat purchased from Harlan, given a choice between two opposite-sex rats: an in-lab-generated F3 rat (UNT, DMSO, EB, A1221) and a purchased rat. A preference score was calculated by subtracting time spent with the F3 rat minus time spent with the Harlan rat, in which positive numbers indicate more time spent near the F3-lineage rat and a negative score indicating time towards the Harlan rat. (**a**) Timeline of PP experiment; (**b**) diagram of experiment on day 3, with the wire zone shaded in gray; preference scores from the wire zone for (**c**) naïve female Choosers with F3-lineage males and (**d**) naïve male Choosers with F3-lineage females. Data were analyzed by linear mixed model (LMM) for effect of F0 treatment within each sex followed by Holm–Sidak pairwise comparisons. LMM estimated marginal means and standard errors are graphed. Males: *n* = 5 UNT, 9 DMSO, 7 EB, 8 A1221; females: *n* = 5 UNT, 10 DMSO, 7 EB, 7 A1221. * = *p* < 0.05 vs. UNT; + = *p* ≤ 0.083 vs. UNT.

The partner preference paradigm was used to determine the extent to which F3 EDC- or control-lineage rats would be preferred (or avoided) to untreated animals in a mating context. An F3 experimental rat (A1221-, EB-, DMSO-lineage or UNT) was placed opposite a naïve rat purchased from Harlan as the Stimulus animals. A separate set of naïve, Harlan-purchased Chooser rats (of the opposite sex) interacted with the stimulus rats through wire mesh dividers held in place by Plexiglas. Similar to the USV experiments, females were gonadally intact and hormone primed to be receptive. A blind experimenter scored the Chooser rats’ behaviors including grooming, rearing, facial investigation of the stimulus rats through the wire mesh and physical contact with the adjacent Plexiglas. As most behaviors occurred in proximity to the stimulus rats, we focused our analysis to the region adjacent to the wire mesh divider (called the wire zone; Figure 6b). The full set of parameters scored within AnyMaze are listed in Supplemental Table S1.

In trials where naïve female Harlan Choosers were exposed to F3 experimental males, linear mixed modeling (LMM) analysis showed that the females’ time spent rearing (*p* = 0.044) and time spent contacting the Plexiglas (*p* = 0.020) were significantly affected by F0 treatment (Figure 6c). Post hoc analysis revealed that Chooser females preferred the EB-lineage males more often than they preferred UNT controls (time rearing, trend *p* = 0.082; time Plexiglas, *p* = 0.030).

When naïve male Harlan Choosers were tested, their total time active (*p* = 0.017), time spent (*p* = 0.009) and number (*p* = 0.022) of rearing bouts, time spent (*p* = 0.014) and number (*p* = 0.026) of bouts contacting the Plexiglas, and number of facial investigation bouts (trend, *p* = 0.075) were affected by F0 treatment (Figure 6d). Post hoc analysis demonstrated that naïve Chooser males avoided EDC-lineage females more frequently than UNT controls (time active UNT vs. A1221 (trend, *p* = 0.063) and UNT vs. EB (*p* = 0.049); rearing number UNT vs. EB (trend, *p* = 0.060); rearing time UNT vs. A1221 (*p* = 0.032) and UNT vs. EB (*p* = 0.025); Plexiglas number UNT vs. EB (*p* = 0.045); Plexiglas time UNT vs. A1221 (trend, *p* = 0.056) and UNT vs. EB ((*p* = 0.032); Figure 6d and Supplemental Table S1).

## 4. Discussion

The current study demonstrates that a transient gestational exposure to estrogenic EDCs can significantly alter behaviorally relevant endpoints for at least three generations. Interestingly, this occurred in a sex- and generation-specific manner. We found modest but significant effects on copulatory behavior in F1 females and F2 males. In the F3 generation, EDC treatment decreased appetitive 50 kHz ultrasonic vocalizations in response to a rat of the opposite sex and affected the preference of EDC-lineage males and females for a naïve conspecific in a mating context. Finally, we found few somatic changes in adulthood. It is interesting that multigenerational effects of EDCs are preferentially manifested, at least in this paradigm, in neurobehavioral rather than somatic outcomes, a result that may relate to the exquisite sensitivity of the brain to developmental hormones and its potential for epigenetic programming [51]. However, it is possible that other somatic or biochemical outcomes not examined here are altered by prenatal EDC exposure, as we only measured a few specific endpoints and only at one timepoint in adulthood. For example, previous studies on prenatal PCB exposure found delays in the timing of puberty in males [52] and transgenerational effects on anogenital index and female sex steroid hormone levels at P60 [36].

A1221 has weakly estrogenic activity but also has other mechanistic actions including through thyroid and aromatase-mediated pathways [53,54]. Thus, while A1221 can produce similar effects to EB, it is not a pure estrogen and will often deviate from the EB group due to its non-estrogenic-mediated actions as shown in this and previous studies [6]. Here, EDC exposure was given on days E16 and E18 of gestation during the period of germline epigenetic changes and the beginning of brain sexual differentiation in the rat. Both processes are vulnerable to environmental perturbations and A1221 exposure at this time can cause epimutations that become embedded in the germline, leading to changes in somatic gene expression in later generations [24] and lifelong alterations in sex-typical reproductive physiology and behavior. Studies have found differences in maternal versus paternal lineage transmission of disease phenotypes as there are many sex differences in germline de- and re-methylation dynamics [55]. In this study, both maternal and paternal lineages were investigated in the F2 generation; however, we did not find any significant lineage effects on our endpoints. Finally, due to experimental constraints we were unable to perform experiments on every lineage combination in the F3 generation and instead selected F3 females of maternal, maternal lineage and F3 males of paternal, paternal lineage. This is an important area of future study.

### 4.1. Transgenerational Somatic Endpoints

Of the somatic changes monitored in the current study, only a few changes were observed in adulthood of EDC-lineage rats, mirroring previous results using this treatment model [36]. Here, we reproduced an increase in body weight at euthanasia in F2-A1221 males. A1221 males had a modest ~7% increase in body weight at euthanasia compared to controls, all given the same ad libitum diet of low-phytoestrogen rat chow. Whether this weight increase is due to increased consumption or a difference in metabolism and energy expenditure between groups should be addressed in future studies. For instance, additional markers of altered metabolism, such as serum insulin or adipokines, could be examined. This finding suggests that PCBs may act according to the “obesogen hypothesis,” in which EDC activity can predispose organisms to obesity and metabolic dysfunction. Future research should investigate the extent to which the transgenerational effects of A1221 can synergistically increase weight gain with a high fat diet in adulthood. In the Mennigen et al. (2018) study [36], both F2 and F3 males with A1221 lineage had increased body weight; however, this was primarily driven through the maternal line and our study used only paternal F3 males. Therefore, this discrepancy is likely due to mechanisms of maternal vs. paternal inheritance.

Increased body weight was also previously found in the female F2- and F3-A1221 littermates [36]; however, there are major differences between these subjects and those in the current study. Here, the F2 females carried a litter to term and were euthanized after weaning, and F3 females were euthanized after the completion of all sociosexual experiments at P120. This resulted in females whose age and postpartum status were vastly different from those in the previous study. Similarly, our finding of an increase in the normalized uterine weight of the F2-A1221 females compared to EB, which was not seen previously, could be due to an interaction between EDC-lineage and postpartum status or might be due to differences in cycle status between the groups. Unfortunately, we did not track the cycle status of the females in the present study, as we presumed that females would be roughly distributed throughout the estrous cycle, and this precludes our ability to rule out cycle effects. Finally, our findings agree with previous work showing that treatment of dams with EDCs on gestational days 16 and 18 do not significantly influence serum testosterone or estradiol concentrations in the F1 and F2 generations [36].

### 4.2. F1 and F2 Generation Adult Mating Behavior

Perturbation of the reproductive axis by estrogenic compounds may affect the expression of sexual behavior in adulthood [56]; thus, we studied the copulatory behavior of the F1 and F2 generations as they were mated to propagate litters for the transgenerational experiment. The timing and setup of the mating trials were designed to replicate the conditions from previous experiments on perinatal EDC treatment in our lab. Therefore, sexually inexperienced EDC-lineage rats were mated to untreated, Harlan-raised rats under non-paced mating conditions.

In the F1 generation, prenatal EDC treatment did not alter male copulatory behavior during their first exposure to sexual experience. While a study using a PCB mixture (PCB 126, 138, 153 and 180) found that prenatal exposure delayed latencies in first and subsequent testing of F1 males [57] our model used a differing PCB mixture that may have differing mechanisms of action. On the other hand, our F1-A1221 females significantly delayed their first lordotic event in response to mounting attempts by a sexually experienced male compared to DMSO. A delay in receptive behavior may indicate a deficiency in copulatory motivation. Similarly, using the same A1221 dose, F1 females in a paced mating paradigm also delayed the pacing of mating encounters and event-return latencies [37] although, in both cases, female lordosis remained intact. Some studies have found EDC effects on lordosis and proceptivity using prenatal endocrine active UV filters [58] or exogenous estradiol [59]. However, other specific PCB mixtures had no influence on female lordosis [57,60] as we found here.

The F2 generation showed a different pattern of results. F2 females had no effects of EDCs in their mating behavior; however, F2 males of EB lineage had a decrease in the mount frequency compared to DMSO. The decrease in the number of mounts did not affect the average intromission ratio, or copulatory efficiency, in which a higher percentage of intromissions to mounts may indicate greater ease to achieve an erection [61]. The decrease is also unlikely to reflect a decreased motivation for sexual activity because the latencies to mount and intromit, better indices of motivation, were not affected. In any case, F2 male mating behavior was not severely impacted by either EDC treatments, at least when comparing the initial sexual event. Future studies should address whether reproductive behavior after repeated sexual experience trials reveals other significant effects.

### 4.3. F3 Generation Adult Sociosexual Behaviors

Ultrasonic vocalizations are emitted by rodents throughout development and are thought to represent affective states and possibly facilitate communication. In adulthood, rat USVs can be characterized by two main types: 22 kHz calls, associated with aversive stimuli, and those in the 50 kHz or above range, associated with arousal states and positive affect [62]. Rats produce a high rate of 50 kHz calls during positive social interactions such as reproductive behavior, juvenile play and tickling by an experimenter. While the 22 kHz calls are emitted by males after ejaculation, the 50 kHz calls are associated with solicitation and copulatory acts [63]. In this study, we used a well-documented paradigm for inducing 50 kHz calls through a brief exposure to a hormonally receptive rat of the opposite sex [40]. Upon removal of the stimulus animal, rats will reliably produce 50 kHz calls. We also added an additional negative control group of untreated rats (UNT) bred in-house alongside our F3 generation. Both negative control groups, UNT and F3-DMSO, behaved similarly. We found a decrease in 50 kHz USV production with EDC lineage, particularly in males. Unfortunately, due to a low *n* per group, our study was underpowered. However, when sexes were combined, we were able to see statistically significant decreases in both EB and A1221 groups compared to DMSO control. As 50 kHz calls appear to facilitate mating interactions by signaling a readiness to mate and orienting the activity of the estrous female [63,64], a decrease in USV calls may indicate a deficit in reproductive fitness.

The 50 kHz calls often display variation in subtype and can be roughly separated into frequency-modulated (FM) or non-FM calls. Although the functional implications are not fully understood for these subtypes, FM calls may signal a dopamine-dependent reward state and are preferentially increased in anticipation of cocaine and amphetamine [41,65]. In contrast, flat calls appear to help coordinate social behavior as they are evoked after separation from cage-mates or potential mates and can induce approach behavior in both mating and non-mating environments [66]. Our findings show a decrease in non-FM calls, which would include the flat subtype, with EB lineage. This may suggest a deficit in the coordination of reproductive behavior instead of a decreased motivation to mate. Interestingly, when F3-A1221 pups were separated from their mother, the rate of neonatal USVs were also decreased in paternal-lineage pups [29], so this effect appears to be consistent throughout development.

In this study, we observed notable sex differences in USV calls, with males calling more frequently and for longer call duration than females. While males are known to emit more 50 kHz calls during rough-and-tumble play behavior than females [67], the two sexes generally produce similar call rates during mating encounters [29,68]. Acquisition of sexual experience and hormonal status of both the experimental and stimulus rats can affect the number of vocalizations [68,69,70]. In naturally cycling females, calls are maximized during proestrus compared to the other cycle states as well as after hormone administration in ovariectomized females [68,71]. The sexually inexperienced females of this study remained ovarian-intact but were supplemented with both estradiol and progesterone to induce the appropriate physiological state. Further, receptivity was confirmed (a lordosis response to an experienced male’s mount) prior to the experiment. Unfortunately, this setup failed to induce vocalizations in the females, while males produced calls at a similar rate to that seen in sexually naïve males in the same paradigm [40]. Future studies should assess female USV production during the appropriate stage of their estrous cycle to determine if calls are increased during their natural behavioral estrus. Thus, while the EDC effects appear to be driven solely by paternal-lineage males, our interpretation of F3 female behavior must take into account that this floor effect may mask further decreases in females USV production.

Finally, we investigated whether F3 rats inherited indicators of reproductive deficits from their EDC ancestry. To test this hypothesis, we allowed naïve Chooser rats to select from an F3 experimental rat or a naïve rat (raised at Harlan), using a partner preference paradigm that previously showed a female preference for F3-vehicle males over F3-vinclozolin males (in that study, males showed no preference for either type of female; [32]). Here, we made the surprising observation of a distinction between our in-house negative controls (UNT and F3-DMSO) and the naïve Harlan stimulus rats, especially when males were choosing between females. While males on average tended to prefer UNT and DMSO females compared to naïve Harlan females, they tended to avoid F3-EB and F3-A1221 females. These results emphasize the importance of negative controls, as environmental factors such as rearing environment (in-house vs. Harlan) can affect behavior. Conversely, when naïve females were Choosers, they showed higher preference scores for F3-EB males than for the UNT controls. This study did not attempt to determine the basis for the differences in choice, although this result is particularly interesting in the context of decreased USV production seen in F3-EB males. Other physical stimuli, such as pheromones, and behavioral cues, also play a role in mate choice, and may outweigh any deficits in social USV calls.

## 5. Conclusions

These results show that prenatal EDC treatment has distinct effects within each generation, in a sexually dimorphic manner, showing the complexity of studying inheritance of EDC exposure. These results extend and complement other data showing transgenerational studies on EDCs as well as other environmental stressors [72] that influence health and disease.

## Figures and Tables

**Table 1:** Summary of significant results.

	Females		Males	
	EB	A1221	EB	A1221
Somatic (F1, F2, F3)				
Body weight	-	-	-	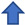 (F2)
Adrenal weight (normalized)	-	-	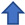 (trend, F1 and F3)	-
Uterine weight (normalized)	-	A1221 > EB (F2)	-	-
**Hormones (F1, F2)—Estradiol (females), Testosterone (males)—n.s.**				
**Mating Behaviors (F1, F2)**				
Mount frequency	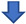 (F2)	-	-	-
Lordosis latency	-	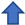 (F1)	-	-
**Partner Preference Behavior (F3)**				
Total time active	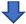	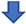 (trend)	-	-
Time rearing	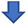	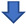	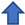 (trend)	-
# Rearing bouts	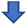 (trend)	-	-	-
Time at Plexiglas	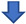	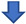 (trend)	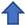	-
# Plexiglas bouts	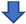	-	-	-
**Ultrasonic Vocalizations (F3)**				
(combined for the sexes)	EB	A1221		
# Total calls	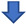	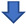		
# Non-FM calls	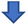			

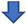
 Decreased compared to DMSO (or UNT for Partner Preference). 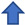 Increased compared to DMSO (or UNT for Partner Preference). FM: Frequency modulated. n.s. and -: No significant effects. #: Number.

**Table 2:** Table of somatic data for each generation.

**F1 MALES**	**DMSO (*n* = 14)**		**EB (*n* = 11)**		**A1221 (*n* = 12)**		
	**Mean**	**±SE**	**Mean**	**±SE**	**Mean**	**±SE**	***p*-Values**
Body Weight (g)	475.2	(±10.9)	462.9	(±12.0)	461.7	(±11.6)	n.s
Norm Adrenal Weight (mg)	0.105	(±2.2 × 10^−3^)	*0.113*	*(±2.6 × 10^−3^)*	0.107	(±2.8 × 10^−3^)	*p* = 0.064
Norm Testes Weight (mg)	8.9	(±0.24)	9.0	(±0.07)	9.3	(±0.25)	n.s.
Serum Testosterone (ng/mL)	1.4	(±0.2)	1.2	(±0.1)	1.1	(±0.2)	n.s.
**F2 MALES**	**DMSO (*n* = 26)**		**EB (*n* = 22)**		**A1221 (*n* = 23)**		
	**Mean**	**±SE**	**Mean**	**±SE**	**Mean**	**±SE**	
Body Weight (g)	449.3	(±5.5)	461.5	(±8.2)	**482.2**	**(±11.4)**	***p* = 0.011**
Norm Adrenal Weight (mg)	0.108	(±1.8 × 10^−3^)	0.111	(±2.3 × 10^−3^)	0.109	(±2.7 × 10^−3^)	n.s.
Norm Testes Weight (mg)	9.3	(±0.009)	9.2	(±0.16)	9.0	(±0.18)	n.s.
Serum Testosterone (ng/mL)	1.1	(±0.1)	1.2	(±0.2)	0.8	(±0.1)	n.s.
**F3 MALES**	**DMSO (*n* = 10)**		**EB (*n* = 9)**		**A1221 (*n* = 9)**		
	**Mean**	**±SE**	**Mean**	**±SE**	**Mean**	**±SE**	
Body Weight (g)	472.4	(±15.2)	464.0	(±12.9)	491.3	(±7.0)	n.s.
Norm Adrenal Weight (mg)	0.108	(±4.7 × 10^−3^)	*0.114*	*(±3.5 × 10^−3^)*	0.100	(±2.7 × 10^−3^)	*p* = 0.076
Norm Testes Weight (mg)	9.1	(±0.27)	9.4	(±0.23)	8.8	(±0.17)	n.s.
**F1 FEMALES**	**DMSO (*n* = 14)**		**EB (*n* = 11)**		**A1221 (*n* = 12)**		
	**Mean**	**±SE**	**Mean**	**±SE**	**Mean**	**±SE**	
Body Weight (g)	286.2	(±6.3)	290.6	(±8.7)	286.0	(±5.4)	n.s.
Norm Adrenal Weight (mg)	0.219	(±9.5 × 10^−3^)	0.205	(±4.3 × 10^−3^)	0.208	(±5.7 × 10^−3^)	n.s.
Norm Ovarian Weight (mg)	0.540	(±2.3 × 10^−2^)	0.528	(±1.9 × 10^−2^)	0.538	(±1.7 × 10^−2^)	n.s.
Norm Uterine Weight (mg)	1.47	(±0.1)	1.99	(±0.4)	1.71	(±0.2)	n.s.
Serum Estradiol (pg/mL)	22.3	(±2.9)	21.8	(±5.4)	19.0	(±2.3)	n.s.
**F2 FEMALES**	**DMSO (*n* = 26)**		**EB (*n* = 22)**		**A1221 (*n* = 23)**		
	**Mean**	**±SE**	**Mean**	**±SE**	**Mean**	**±SE**	
Body Weight (g)	283.0	(±3.4)	291.4	(±4.6)	289.6	(±3.7)	n.s.
Norm Adrenal Weight (mg)	0.208	(±3.9 × 10^−3^)	0.197	(±4.9 × 10^−3^)	0.203	(±4.0 × 10^−3^)	n.s.
Norm Ovarian Weight (mg)	0.540	(±1.5 × 10^−2^)	0.555	(±1.7 × 10^−2^)	0.547	(±1.3 × 10^−2^)	n.s.
Norm Uterine Weight (mg)	1.63	(±0.1)	1.45	(±0.1)	**1.97**	(±0.2) *	*p* = 0.04
Serum Estradiol (pg/mL)	15.4	(±1.6)	14.4	(±1.3)	22.2	(±3.4)	n.s.
**F3 FEMALES**	**DMSO (*n* = 11)**		**EB (*n* = 7)**		**A1221 (*n* = 9)**		
	**Mean**	**±SE**	**Mean**	**±SE**	**Mean**	**±SE**	
Body Weight (g)	285.8	(±7.1)	273.3	(±8.4)	287.4	(±8.3)	n.s.
Norm Adrenal Weight (mg)	0.202	(±6.1 × 10^−3^)	0.208	(±8.3 × 10^−3^)	0.207	(±7.4 × 10^−3^)	n.s.
Norm Ovarian Weight (mg)	0.437	(±4.4 × 10^−2^)	0.447	(±1.8 × 10^−2^)	0.475	(±3.1 × 10^−2^)	n.s.
Norm Uterine Weight (mg)	2.97	(±0.5)	2.33	(±0.3)	4.49	(±1.2)	n.s.

Body weights are shown for the day of euthanasia, with adrenal, ovarian, uterine, and testicular weights measured postmortem, and the ANOVA *p*-value for a main effect of Treatment. Norm: normalized to body weight. SE: standard error of the mean. n.s.: No significant effects. Bold text indicates significantly different (*p* < 0.05) from DMSO, and italicized text indicates a trend (0.05 < *p* < 0.1) from DMSO in post hoc comparisons. *, A1221 significantly different from EB (*p* = 0.04).

**Table 3:** Ultrasonic vocalization parameters for F3 males and females.

**MALES**	**UNT (*n* = 5)**		**DMSO (*n* = 10)**		**EB (*n* = 6)**		**A1221 (*n* = 6)**		***p*** **-Values**	**(Sex-Combined)**
	**Mean**	**±SE**	**Mean**	**±SE**	**Mean**	**±SE**	**Mean**	**±SE**	**Treatment**	**Sex**
Number of total calls	101.2	(±23.6)	124.5	(±28.3)	***33.5***	***(±12.3)***	68.8	(±7.8)	***p* < 0.001**	***p* < 0.001**
Number of non-FM calls	48.2	(±10.5)	62.8	(±13.9)	**22.8**	**(±7.5)**	36.5	(±10.1)	***p* = 0.024**	***p* < 0.001**
Number of FM calls	53.0	(±14.5)	47.2	(±11.5)	17.4	(±6.2)	49.5	(±9.4)	n.s.	***p* < 0.001**
Percentage of FM calls	50.3	(±4.8)	47.3	(±3.8)	46.2	(±7.1)	59.7	(±4.8)	n.s.	n.s.
Average call duration (ms)	1.44	(±0.22)	1.41	(±0.13)	1.13	(±0.13)	1.61	(±0.19)	n.s.	***p* = 0.032**
**FEMALES**	**UNT (*n* = 5)**		**DMSO (*n* = 9)**		**EB (*n* = 6)**		**A1221 (*n* = 8)**			
	**Mean**	**±SE**	**Mean**	**±SE**	**Mean**	**±SE**	**Mean**	**±SE**		
Number of total calls	10.0	(±2.1)	21.4	(±10.4)	13.8	(±6.7)	7.3	(±4.5)		
Number of non-FM calls	5.8	(±1.5)	10.7	(±4.0)	8.8	(±4.1)	9.6	(±5.0)		
Number of FM calls	4.2	(±0.7)	26.5	(±12.3)	7.8	(±3.6)	4.3	(±2.8)		
Percentage of FM calls	45.0	(±5.7)	40.5	(±10.0)	55.4	(±12.4)	32.0	(±11.8)		
Average call duration (ms)	0.80	(±0.10)	1.06	(±0.22)	1.19	(±0.20)	1.28	(±0.26)		

Two-way ANOVA *p*-values for a main effect of Treatment and Sex are provided for the sexes combined (shown next to the male data, but applicable to both sexes). Bold text indicates significantly different at *p* < 0.05 from DMSO in post hoc pairwise comparisons within each sex, and italicized text indicates a trend (0.05 < *p* < 0.1). n.s.: No significant effects, FM: frequency modulated.

## Data Availability

Data will be made available upon request.

## Supplementary Materials

The following supporting information can be downloaded at: https://www.mdpi.com/article/10.3390/toxics10020047/s1, Table S1: Table of estimated marginal means and standard errors for F3 partner preference scores.
